# Proving of Lac Caninum

**Published:** 1889-08

**Authors:** S. Swan


### PROVING OF LAC CANINUM.

S. Swan, M. D.

January 17th, 1871.—Being in usual good health, commenced at eleven A. M. to take drop doses of 33d centesimal potency every hour. At seven P. M., pain over left eye in organs of size and weight. Sensations of coryza, principally in left nostril; thin mucus passing down posterior nares.

January 21st.—The proving having been interrupted during 18th and 19th, was resumed to-day. Great redness, with mottled appearance on inner side of lower lip, with white ulcerated spots and great sensitiveness. Aching pain round waist in a line with kidneys. Slight nausea. At night, on retiring, dryness of throat; pain over outer angle of right eye.

January 22d.—Fluent coryza from left nostril in morning when in the wind.

